# Targeting long non-coding RNA MALAT1 reverses cancerous phenotypes of breast cancer cells through microRNA-561-3p/TOP2A axis

**DOI:** 10.1038/s41598-023-35639-x

**Published:** 2023-05-27

**Authors:** Sara Hajibabaei, Nahid Nafissi, Yasamin Azimi, Reza Mahdian, Fatemeh Rahimi-Jamnani, Vahideh Valizadeh, Mohammad Hessam Rafiee, Masoumeh Azizi

**Affiliations:** 1 Molecular Medicine Department, Biotechnology Research Center, Pasteur Institute of Iran, 69th Pasteur Street, Kargar Avenue, Tehran, Iran; 2 Breast Surgery Department, Iran University of Medical Sciences, Tehran, Iran; 3 Department of Mycobacteriology and Pulmonary Research, Pasteur Institute of Iran, Tehran, Iran; 4 Department of Nano-Biotechnology, New Technologies Research Group, Pasteur Institute of Iran, Tehran, Iran; 5 Blood Transfusion Research Center, High Institute for Research and Education in Transfusion Medicine, Tehran, Iran

**Keywords:** Cancer, Cell biology, Genetics, Immunology, Molecular biology, Biomarkers, Diseases, Oncology

## Abstract

Non-coding RNAs, including Inc-RNA and miRNA, have been reported to regulate gene expression and are associated with cancer progression. MicroRNA-561-3p (miR-561-3p), as a tumor suppressor, has been reported to play a role in preventing cancer cell progression, and MALAT1 (Lnc-RNA) have also been demonstrated to promote malignancy in various cancers, such as breast cancer (BC). In this study, we aimed to determine the correlation between miR-561-3p and MALAT1 and their roles in breast cancer progression. The expression of MALAT1, mir-561-3p, and topoisomerase alpha 2 (TOP2A) as a target of miR-561-3p was determined in BC clinical samples and cell lines via qRT-PCR. The binding site between MALAT1, miR-561-3p, and TOP2A was investigated by performing the dual luciferase reporter assay. MALAT1 was knocked down by siRNA, and cell proliferation, apoptotic assays, and cell cycle arrest were evaluated. MALAT1 and TOP2A were significantly upregulated, while mir-561-3p expression was downregulated in BC samples and cell lines. MALAT1 knockdown significantly increased miR-561-3p expression, which was meaningfully inverted by co-transfection with the miR 561-3p inhibitor. Furthermore, the knockdown of MALAT1 by siRNA inhibited proliferation, induced apoptosis, and arrested the cell cycle at the G1 phase in BC cells. Notably, the mechanistic investigation revealed that MALAT1 predominantly acted as a competing endogenous RNA in BC by regulating the miR-561-3p/TOP2A axis. Based on our results, MALAT1 upregulation in BC may function as a tumor promoter in BC via directly sponging miRNA 561-3p, and MALAT1 knockdown serves a vital antitumor role in BC cell progression through the miR-561-3p/TOP2A axis.

## Introduction

Breast cancer is an urgent global priority. This disease is the most common cancer in women, accounting for 30% of newly reported cases, and is one of the leading causes of cancer deaths^1^. BC is generated via one-of-a-kind elements like age, menarche history, reproductive patterns, bodily activity, breast traits, and physique habitus. Although tremendous advances have been made in the early detection and therapeutics of breast cancer over the last 20 years, the disease remains a significant public health problem^2^. In this context, novel therapeutic techniques for BC are in pressing need. We undertook a long non-coding RNA (LncRNA)-based method to apprehend the underlying mechanism in BC development and enhance novel intervention strategies.

LncRNA genes are a significant proportion of non-coding RNAs that play a vital role in normal growth and also in the tumorigenesis process^3^. It is estimated that the human genome contains 23,000 LncRNA genes, which is more than 20,000 protein-coding genes^4^. Their physiological and pathological functions are mediated by their interactions with microRNAs, mRNAs, proteins, and genomic DNA^5^. Since the deregulation of gene expression is an important event in carcinogenesis, it has been suggested that a significant proportion of cancer risk may be attributed to transcribed LncRNAs from cancer-related loci^6^.

Dysregulation and deficiency, or mutations of LncRNAs, have been reported in complex diseases^7–9^, including BC cancer^10^. Depending on their functions and pattern of expression, they can be divided into tumor suppressors and oncogene classes^11^. In addition, growing exploratory evidence supports the idea that LncRNAs function as competitive endogenous RNAs (ceRNAs), which compete for microRNA (miRNA) binding to their upregulated target genes^12^. The ceRNA theory can critically help to understand and identify the function of lncRNA^13^. In the case of BC, the LncRNA expression profile is often dysregulated, and numerous LncRNAs have been concerned in BC tumorigenesis, such as LINC01133, ZEB1-AS1, and ABHD11-AS1^14^. Due to the improvement of LncRNA-based therapeutic approaches, defining the function of exclusive LncRNAs in a tumorigenic manner is of first-rate importance. Hence, in this study, we intend to elucidate the oncogenic role of LncRNA MALAT1 in BC, which acts as a miR-561 sponge.

Metastasis-Associated Lung Adenocarcinoma Transcript 1 (MALAT1) is a nuclear-enriched long non-coding RNA generally overexpressed in patient tumors and metastases. Overexpression of MALAT1 is positively correlated with tumor progression and metastasis in many tumor types, including breast tumors. MALAT1 was initially identified as a lncRNA whose expression was increased in early human non-small cell lung tumors that were more prone to metastasis. Subsequently, MALAT1 is highly expressed in numerous other human cancers, including, but not limited to, breast, lung, ovarian, prostate, cervical, endometrial, colorectal, gastric, pancreatic, sarcoma, bladder, brain, hepatocellular carcinoma, esophageal squamous cell carcinoma, multiple myeloma, renal cell carcinoma, and lymphoma^15,16^. As a ceRNA, MALAT1 can interact with miRNAs (miR-205, miR-1297, miR-217, and miR-155), ultimately driving changes in cellular phenotypes such as invasion, metastasis, proliferation, migration, and apoptosis^17^. In addition, MALAT1 may affect cancer carcinogenesis by activating the Wnt/-catenin, ERK/MAPK, and PI3K/AKT pathways, while concomitant activation of oncogenic pathways may cause strong carcinogenic effects^18^.

Topoisomerase alpha 2 (TOP2A), a gene located in 17 q12–21 and consisting of two subunits, is a marker of proliferation and chemotherapy resistance in various cancer types, including adrenocortical carcinoma and breast carcinoma. In addition, several miRNAs have been reported to play a regulatory role by directly inhibiting the target TOP2A in cancer^19,20^.

In this study, MALAT1 overexpression was detected in BC tissues and cells. Loss-of-function experiments suggest that the silencing of MALAT1 inhibits BC proliferation and invasion. In addition, the primary mechanism of interaction between MALAT1 and miR-561 has been identified. We found that MALAT1 can cause miR-561 to "sponge" information and impair TOP2A mRNA degradation (as a target of miR-561) by preventing its association with TOP2A mRNA targeting.

## Results

### MALAT1 and TOP2A are upregulated in BC tissues and cell lines

**Figure 1:**
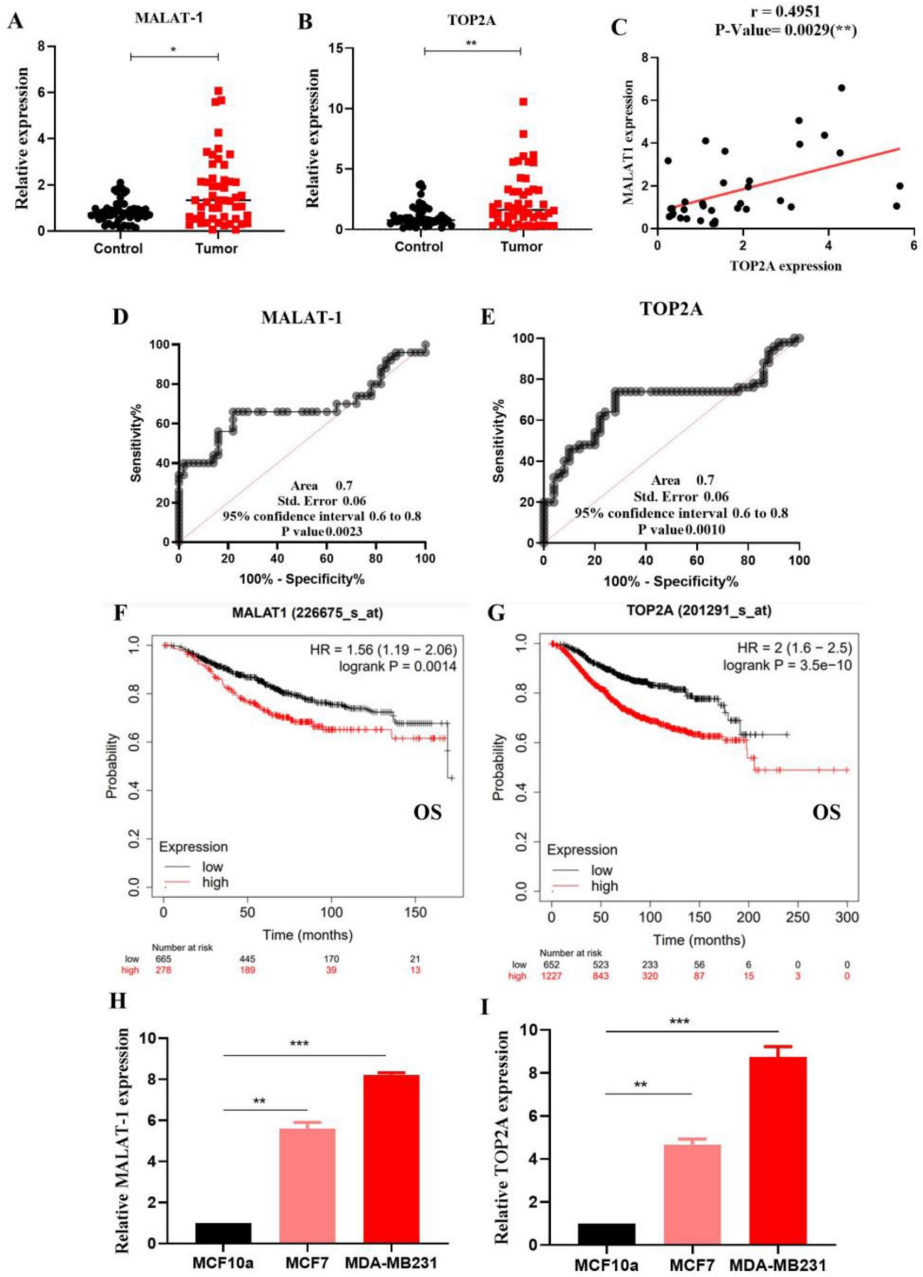
LncRNAs MALAT1 and TOP2A showed different expression patterns in BC patients (n = 50) and cell lines. Levels of MALAT1 and TOP2A significantly increased (**A**, **B**) in patients with BC as compared with normal samples using qRT-PCR. Also, increased expression of TOP2A was directly correlated with MALAT1's increased level of expression (r = 0.4951) (**C**). Area under the curve (AUC) and receiver operating characteristics (ROC) for MALAT1 and TOP2A (**D**, **E**). The Kaplan–Meier plotter for overall survival shows the prognostic role of MALAT1 and TOP2A mRNA expression in breast cancer (**F**, **G**). MALAT1 and TOP2A expression in BC cell lines as compared with the normal sample (MCF10a) (**H**); ***P 0.001; **P 0.01; *P 0.05; mean ± SD.

The LncRNA expression signatures of BC were investigated based on 50 pairs of BC tissues and adjacent normal tissues. The results of differential expression analysis of MALAT1 and TOP2A in BC showed that the expression of MALAT1 and TOP2A in tumor tissues increased significantly in patients' tissues compared to normal tissues (Fig. 1A, B). The expression of these genes was assessed based on the breast cancer TCGA database to assess the prognostic significance of MALAT1 and TOP2A in breast cancers (https://ualcan.path.uab.edu).

The database found breast cancer tumors had much higher expression than normal tissue (Supplementary file). The above results indicated that TOP2A up-regulation might be related to MALAT1. Therefore, we investigated the relationship between MALAT1 and TOP2A expression in BC patients. According to the results of the correlation analysis, we found that MALAT1 was significantly positively associated with TOP2A expression in BC tissues (r = 0.4951; P value = 0.0029) (Fig. 1C). We also determined the predictive value of these altered MALAT1 and TOP2A expression levels using ROC curves (area under the curve, AUC = 0.7) (Fig. 1D, E). As shown in Table 1, increased MALAT1 expression in BC tissue was significantly associated with lymphatic involvement. An online KM plotter database (http://kmplot.com/analysis/) using microarray data showed that Lnc-MALAT1 and TOP2A expression was significantly correlated with OS (overall survival) in 1880 breast cancer patients (HR = 1.56, P = 0.0014, and HR = 2, P = 3.5 e−10, respectively) (Fig. 1F,G). As shown in Fig. 1H, I, MALAT1, and TOP2A were more upregulated in MDA-MB231 and MCF7 cells compared with the normal breast tissue cell line (MCF10a).

**Table 1:** Association between MALAT1 expression and clinicopathologic features of breast cancer.

Characteristics	No	High expression of MALAT1 (%)	P value
Age			
> 45	29	17 (42.0)	Ns
≤ 45	21	13 (51.8)	
Estrogen receptor status			
ER+	22	12 (52.1)	Ns
ER−	28	18 (50.0)	
Tumor size (cm)			
≤ 2	23	13 (45.0)	Ns
> 2	27	17 (51.5)	
Her2/neu receptor status			
Her2/*neu*+	23	15 (53.8)	Ns
Her2/*neu*−	27	15 (47.2)	
Histologic subtype			
Ductal	27	17 (44.7)	Ns
Lobular	23	13 (57.6)	
Lymph node involvement			
Negative	24	11 (34.0)	*P < 0.05
Positive	26	23 (60.8)	
Stage			
I/II	23	15 (43.0)	Ns
III	27	15 (57.9)	
Progesterone receptor status			
PR+	23	13 (51.4)	Ns
PR−	27	17 (48.8)	

*Ns* non-significant. *: significant

### MALAT1 regulates the expression of TOP2A via competitive binding with miR-561-3p in BC

**Figure 2:**
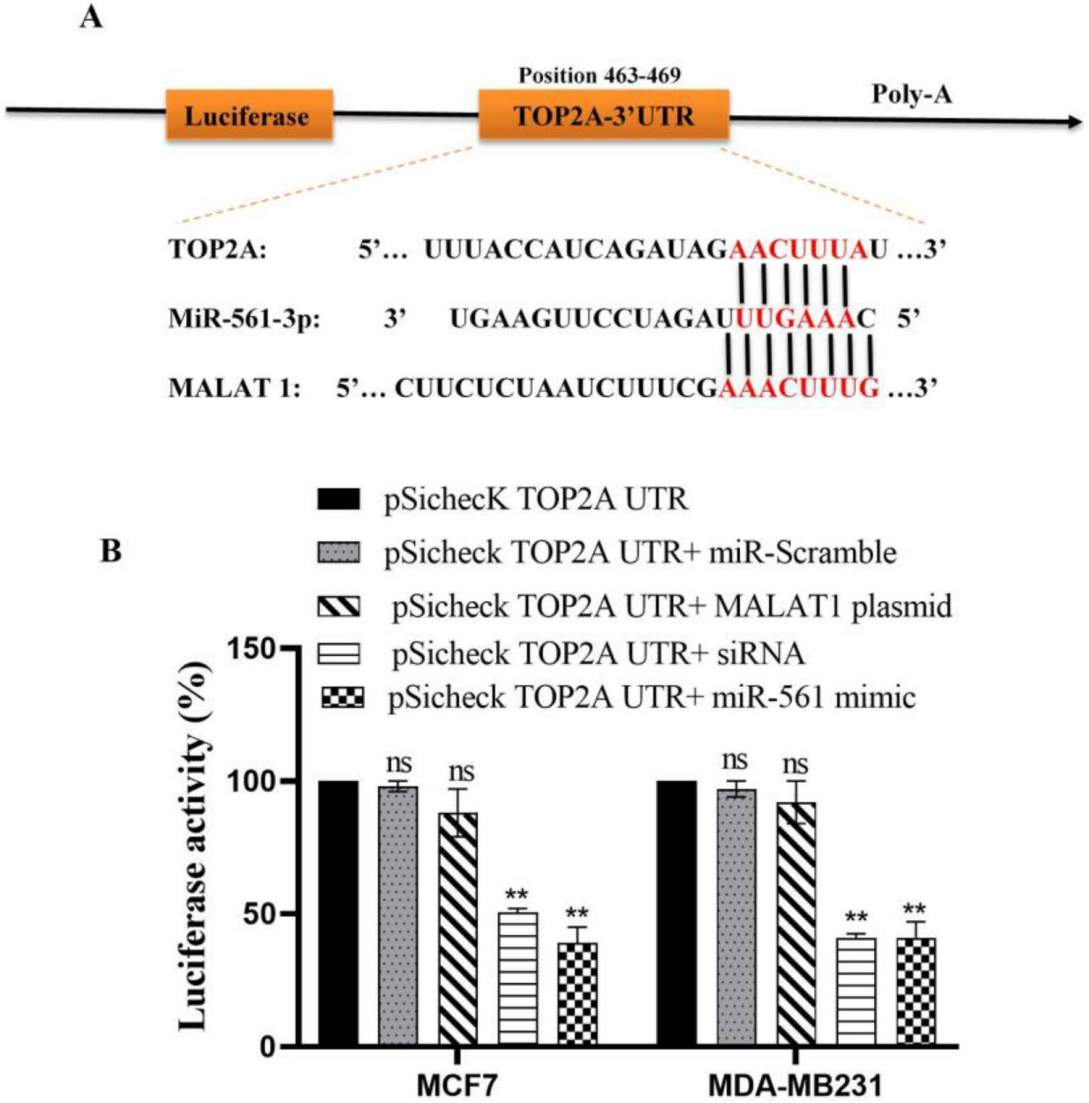
TOP2A and MALAT1 target miR‑561 in BC. Prediction of binding sites between miR‑561, TOP2A, and MALAT1 (**A**). Dual‑luciferase reporter assay indicated that siRNA-MALAT1 and miR‑561 mimics could decrease the luciferase activity of reporter in BC cells (**B**), **P 0.01. Mean ± SD.

We initially identified the regulation of TOP2A by miR-561 to investigate the impact of MALAT1 on TOP2A expression. The Dual-Luciferase Reporter Assay showed that miR-561 mimics decreased the luciferase activity of reporters with the 3′ UTR of TOP2A. Figure 2A shows that miR-561 has a binding site in the 3′ UTR of TOP2A mRNA. However, miR-561 did not affect the luciferase activity of the reporter containing the mutant 3′ UTR of TOP2A (Fig. 2B).

We discovered that MALAT1 has miR-561-3p binding sequences using an in silico study. In BC cells transfected with miR-561 mimic and the reporter vector MALAT1-WT but not MALAT1-Mut, the relative luciferase activities were reduced. A luciferase assay was carried out in MCF7 and MDA-MB-231 cells that had been transfected with a reporter plasmid containing the 3′-UTR of TOP2A and miR-561 mimic, miR-561 mimic + MALAT1 plasmid, or miR-561 mimic + mutant MALAT1 plasmid to determine the influence of MALAT1 on TOP2A expression. Cells transfected with the miR-561 mimic displayed significantly less luciferase activity than control cells. In contrast, the MALAT1 plasmid overrode this regulation and boosted the activity of the luciferase gene in cells transfected with the miR-561 mimic and the MALAT1 plasmid. These findings suggest that MALAT1 may control TOP2A expression by sponging miR-561.

### Evaluation of miR-561 for prognosis in breast cancer and effective investigation of its target genes

**Figure 3:**
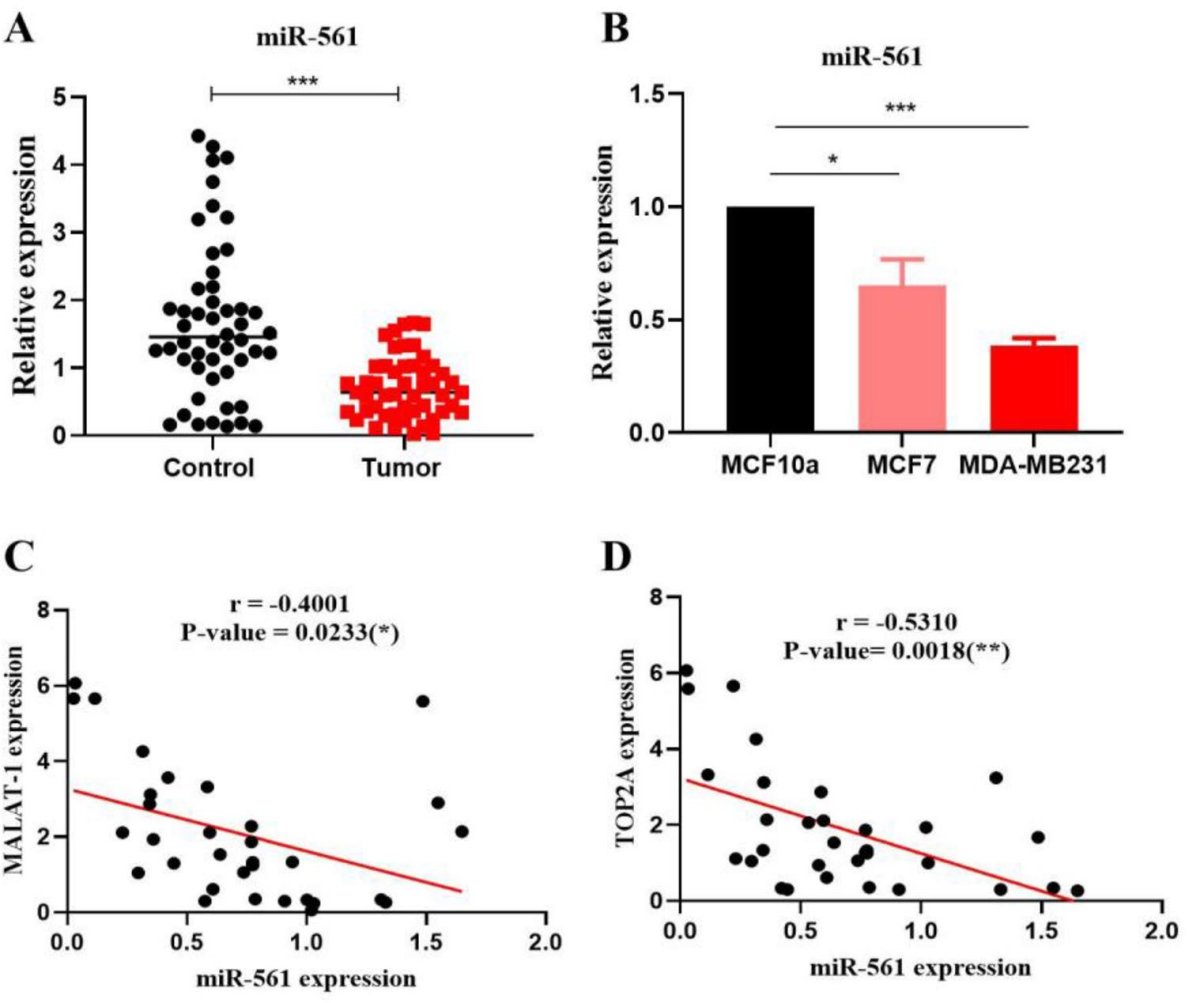
MiR‐561 expressions were analyzed in BC tissue samples and cell lines. MiR‐561 expression was downregulated in BC tissues as compared with normal samples using qRT‐PCR (**A**). Expression of miR-561 in BC cell lines compared to normal breast tissue samples (**B**). Decreased expression of miR-561 was correlated with MALAT1 and TOP2A mRNA levels (**C**, **D**); ***P 0.001; **P 0.01; ns: non-significant.

In this study, miR-561 was significantly down-regulated in breast cancer tissue compared with noncancerous tissue (Fig. 3A). Based on the breast cancer TCGA miRNA database, miR-561 expression was measured to determine its prognostic value in breast malignancies. In the database, breast cancer tumors (n = 749) had significantly less expression than normal tissue (n = 76) (P = 1.975 e−05; Supplementary file). As shown in Fig. 3B, miR-561 was more downregulated in breast cancer cell lines with the highest expression level of PD-L1 compared with a normal breast tissue cell line (MCF10a). As compared with noncancerous samples with low MALAT1 and TOP2A levels, BC cases with high MALAT1 and TOP2A levels showed low miR-561 levels, with Pearson correlation analyses revealing a clear inverse relation between miR-561 expression and MALAT1 and TOP2A mRNA levels (r = 0.4001; P value = 0.0233; r = 0.5310; P value = 0.0018, respectively; Fig. 3C, D).

### Knockdown of MALAT1 leads to an increase in miR-561 expression and downregulation of TOP2A.

**Figure 4:**
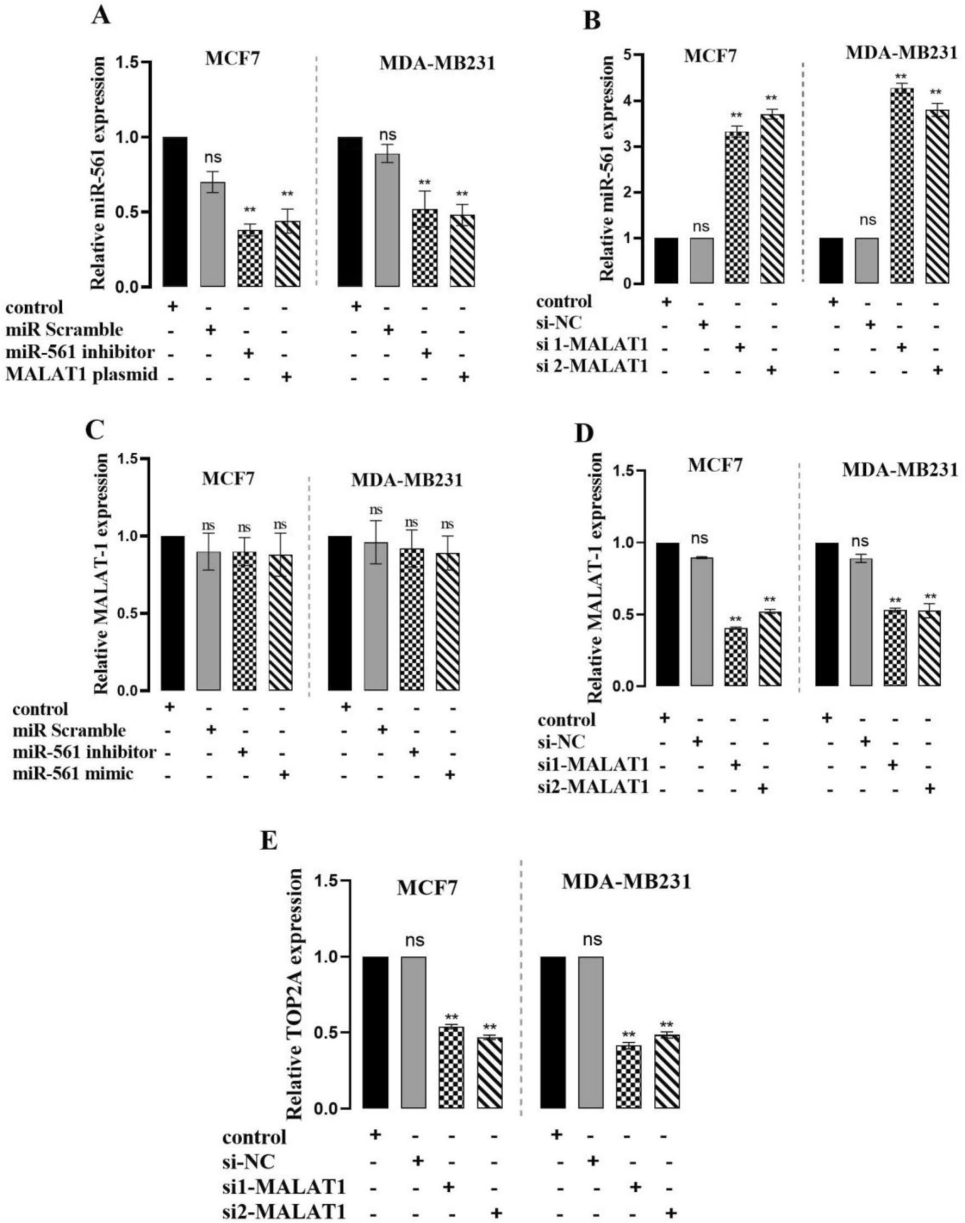
MALAT1 upregulated TOP2A expression by repressing miR-561-3p in BC cells. MCF7 and MDA-MB231 cells were transfected with MALAT1-plasmid or with a miR-561 inhibitor as the positive control. After 24 h, the mRNA level of miR-561 was significantly lower in BC cells transfected with MALAT1-plasmid or with a miR-561 inhibitor than that in control cells (**A**). BC cells were transfected with MALAT1-siRNA or with si-NC as the negative control. After 24 h, the mRNA level of miR-561 in BC cells transfected with MALAT1-siRNA was significantly higher than in si-NC-transfected cells (**B**). MCF7 and MDA-MB231 cells were transfected with miR-561 mimics, inhibitors, or the corresponding controls. MiR-561 up- or down-regulation did not affect the expression of MALAT1 in transfected cells (**C**). MCF7 and MDA-MB231 cells were transfected with MALAT1-siRNA or with siRNA-NC as the negative control. MALAT1 and TOP2A mRNA levels were significantly lower in BC cells transfected with MALAT1-siRNA than in si-NC-transfected cells after 24 h (**D**, **E**). ***P 0.001; **P 0.01; *ns* non-significant; mean ± SD.

To confirm the ceRNA network between MALAT1, miR-561, and TOP2A, we inhibited the expression of MALAT1, and it showed the expression of TOP2A was downregulated, which was consistent with cells treated by miR-561 mimics. RT-qPCR was carried out to quantify miR-561 expression in BC cells transfected with either si-MALAT1 or si-NC. The expression of miR-561 was dramatically higher when MALAT1 was knocked down in BC cells (Fig. 4A–E, *P < 0.05).

### Silencing of MALAT1 restrains breast cancer proliferation and stimulates apoptosis

**Figure 5:**
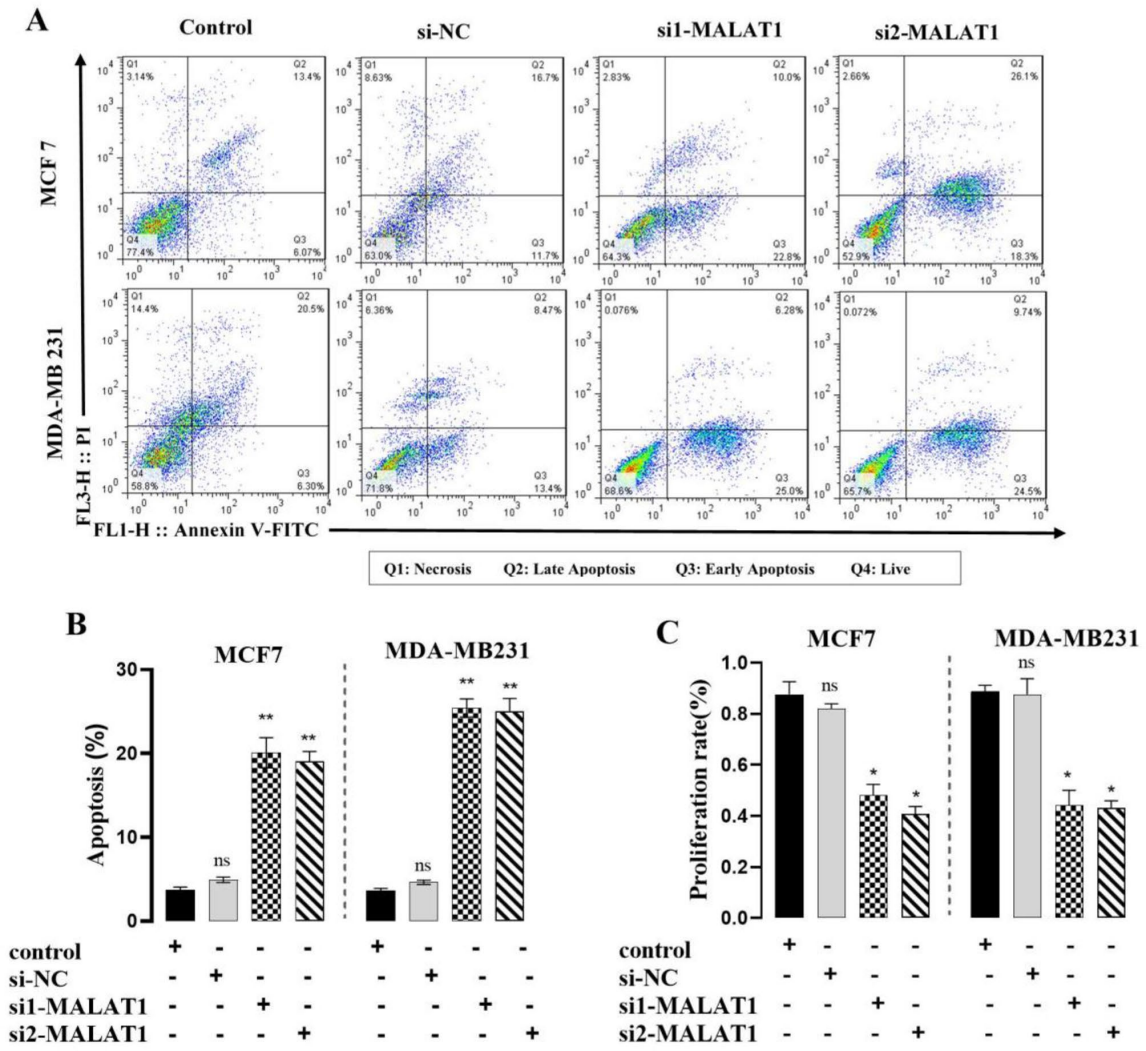
MALAT1 inhibits proliferation and stimulates apoptosis. Flow cytometry analysis of apoptosis in BC siRNA-transfected cells. Compared to the control cells, the treatment of MCF-7 and MDA-MB231 cells with si-MALAT1 showed a significant increase in apoptosis (**A**, **B**). The analysis of cell proliferation in BC cells with si1-MALAT1 and si2-MALAT1 transfection. The MTT assay was done each day for 3 consecutive days (**C**). ***P 0.001; **P 0.01; *P 0.05; *ns* non-significant; mean ± SD (n = 3).

In contrast to MCF10a breast epithelial cells, MALAT1 expression was higher in BC cells. We used siRNA technology to carry out knockdown experiments in both BC cells expressing endogenous MALAT1 at various levels to ascertain the biological role of MALAT1 in BC. MALAT1 was significantly down-regulated after transfection with siRNA that targets MALAT1. Annexin-V/propidium iodide (PI) double labeling provided additional evidence that MALAT1 knockdown significantly increased apoptosis in MCF-7 and MDA MB-231 cells as compared to control cells (Fig. 5A, B). MALAT1 knockdown dramatically decreased the growth of BC cells, according to the MTT experiment. (Fig. 5C).

### MALAT1 knockdown stops the cell cycle in BC cells at the G0/G1 phase

**Figure 6:**
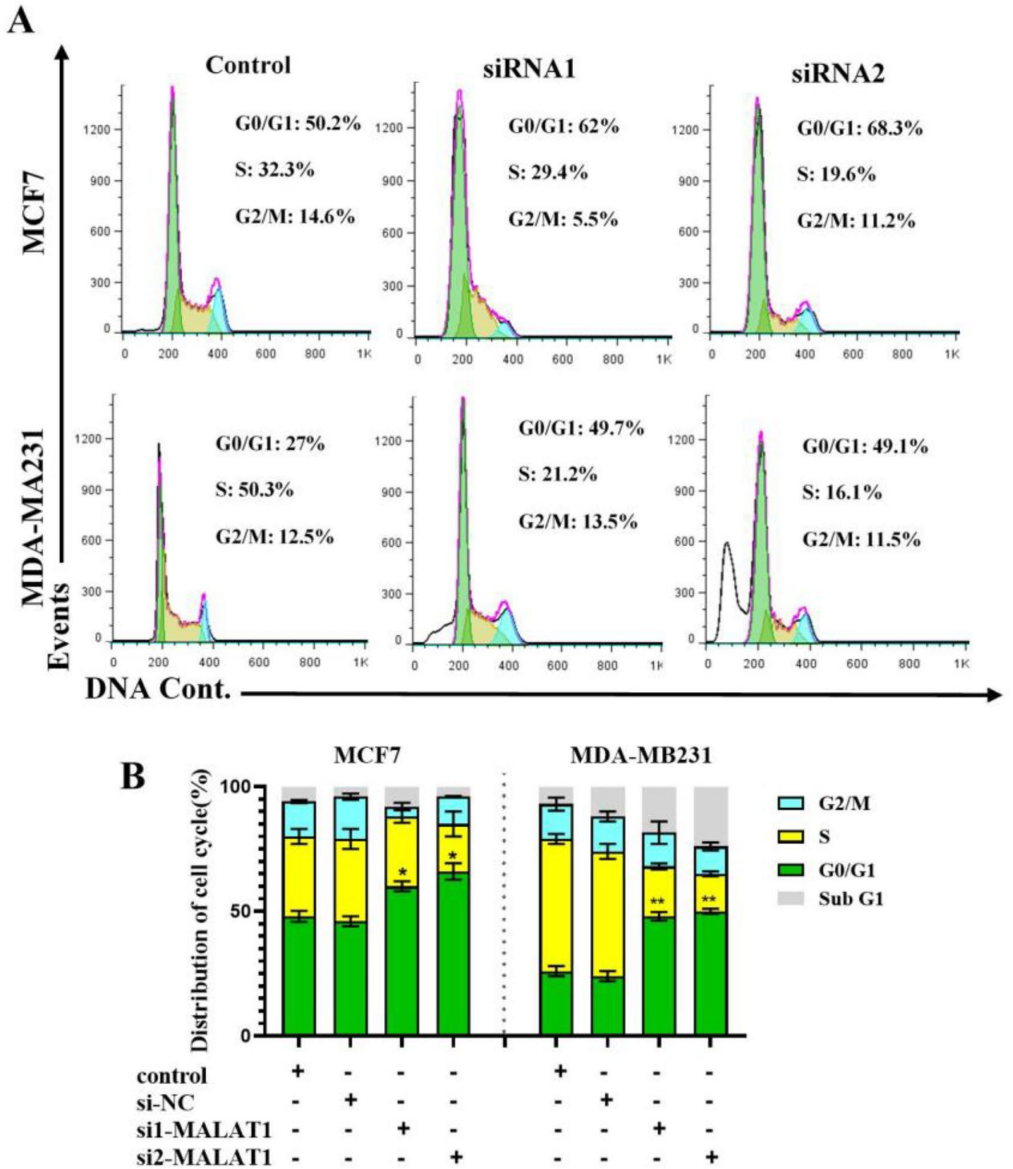
MiR‐561 effect on BC cell cycle at the G0/G1 phase. MCF-7 and MDA-MB 231 cells were transfected with si-MALAT1 and subjected to PI staining before flow cytometry (**A**). The table (bottom) shows the percentage of cells in each cell cycle phase (**B**). **P 0.01. *P 0.05; mean ± SD.

Next, we analyzed the impact of the knockdown of MALAT1 on cell cycle distribution and apoptosis. Flow cytometric analysis revealed a significant increase in the percentage of the G0/G1-phase cells and a decrease in the percentage of the S-phase cells in MALAT1-depleted BC cells (Fig. 6A, B). These data imply that MALAT1 is involved in BC cell proliferation and survival.

## Discussion

Breast cancer has a high survival rate as the most prevalent malignant cancer and the leading cause of death in women, yet lowering its incidence and mortality is still a top concern for society^21^. Additionally, it has been shown that LncRNAs have a significant role in the prognosis of cancers since they are unregulated in several tumors and regulate pathways relevant to cancer. Numerous LncRNAs are directly related to tumor cell growth, development, and metastasis^22^.

The expression of tissue-specific LncRNA makes them exciting candidates for developing diagnostic markers or potential therapeutic targets for systemic treatment. The ability to target LncRNAs at different functional levels offers a wide range of therapeutic options. Targeted approaches may include DNA-based drugs and small-molecule inhibitors^23,24^. A previous study showed that MALAT1 LncRNA promotes BC progression and functions as a ceRNA to regulate DUSP7 expression by sponging miR-155-5p^25^. Consistent with these findings in mice, it has been shown that high levels of MALAT1 in BC are associated with increased tumor size and stage and poor prognosis in human patients^26,27^.

In this study, we identified a crucial BC-associated LncRNA, MALAT1. Also known as nuclear enrichment autosomal transcript 2 (NEAT2), MALAT1 was initially recognized through subtractive hybridization as one of the transcripts most significantly overexpressed in metastatic non-small cell lung cancer tissues^16^. Human MALAT1 RNA consists of a single exon of ~ 7 kb without an open reading frame for protein-coding. Unlike most LncRNAs, MALAT1 is evolutionarily very well conserved and highly expressed in normal tissues^28^. However, the relationship between MALAT1 and BC needs to be further studied. The expression pattern and regulatory mechanism of MALAT1 have been reported in melanoma^29^, lung cancer^30^, prostate cancer^31^, and BC^32^.

In our investigation, tumor tissues had much higher levels of MALAT1 expression than the adjacent non-cancerous tissues. We hypothesized that MALAT1 is upregulated in BC and may be regarded as an oncogene in carcinogenesis based on the results of qRT-PCR LncRNA expression. Furthermore, poor overall survival and advanced clinical characteristics were linked to increased MALAT1 expression.

According to Miao et al.^33^ and our findings, MALAT1 expression in BC tissue was significantly linked to lymphatic formation. MALAT1 knockdown dramatically decreased TNBC cell migration, invasion, and arrest in the G0/G1 phase, according to in vitro experiments^34^. Our findings thus suggest that MALAT1 may play a tumor-promoting role in the development of TNBC tumors. However, more research is required to fully understand how MALAT1 operates in TNBC.

By sponging miR-101b (position 31), MALAT1 regulates Rac1 expression. MALAT1 can also regulate TGF-expression by targeting miR-376a, which promotes osteosarcoma development^35^. Furthermore, MALAT1 promotes BC progression and doxorubicin resistance via regulating miR-570^32^. In our study, we discovered that MALAT1's effects on BC cell progression were mediated by miR-561-3p. Our data showed that MALAT1 is competitively bound to miR-561-3p, reducing the inhibition of its target genes like TOP2A. This leads to an increase in the proliferation of BC cells. Expression of miR-561-3p was down-regulated and negatively correlated with MALAT1 expression in BC tissues. MALAT1 suppression resulted in significant upregulation of miR-561–3p expression and then downregulation of TOP2A as a miR-561 target. Besides, our study also found a binding relationship between miR-561 and TOP2A in a dual-luciferase reporter gene assay. Highly expressed TOP2A is involved in many cancers, with its expression estimated to be elevated or deregulated in the majority of human cancers^36^.

Through post-transcriptional regulation^37^, processing, mRNA splicing^38^, translation, or post-translational protein modification, long non-coding RNAs can also influence cell homeostasis. Additionally, it influences mRNA through the binding of microRNA, which lowers the amount of short free RNA and lessens the impact on transcript coding^39,40^. The balance of metabolic activities is necessary for proper cell function. According to studies, LncRNAs play a key role in cellular metabolism, especially in cancer cells. For example, in gallbladder cancer^41^, LncRNA-PAGBC binds to the tumor-suppressor microRNAs miR-133b and miR-511 in a competitive manner, titrating the miRNAs from the SOX4 and PIK3R3 binding sites and activating the AKT/mTOR signaling cascade.

The results of our study disclosed that MALAT1 could serve as a sponge for miR-561 in BC, and miR-561 is recognized as a tumor suppressor based on previously published research. MiR-561 in glioblastoma multiforme (GBM) can suppress U87 development and proliferation through c-myc regulation, making it a useful model for the treatment of the condition^42^. In our study, miR-561's role as a tumor suppressor, which inhibits BC cell proliferation and metastasis, was also confirmed. In human BC tissues, miR-561 and MALAT1 also had a negative correlation. Rescue tests further demonstrated that miR-561 could partially reverse the overexpression or depletion of MALAT1 in BC cells. Such data demonstrated that MALAT1 might sponge miR-561 to enhance the development of BC tumors.

Our study supported the hypothesis that LncRNA-MALAT1 competitively binds to miR561-3p and promotes BC cell proliferation and metastasis by overactivating TOP2A. Targeted treatment of tumors is now widely accepted. The results of this study may provide new insights into BC's molecular therapy. In the future, we will further investigate the mechanism of other targets of lncRNA MALAT1 and the role of these targets in BC. While our results provide therapeutic implications for BC treatment, experimental results and their effective application in clinical practice need further validation.

## Methods

### Patients and cell lines

A total of 50 BC tissue samples and matched normal tissue samples were collected from the Khatam al-Anbia hospital. The university’s ethics committee approved this work after receiving the written informed consent of all participants. Table 1 provides detailed information for each tissue donor. Two human BC cell lines (MCF-7 and MDA-MB-231) and a breast normal cell line (MCF-10a) were purchased from the Cell Bank of the Pasteur Institute of Iran, Tehran, Iran. Cell lines were cultured in DMEM-F12 supplemented with 10% fetal bovine serum (FBS), 100 μL/mL penicillin, and 100 mg/mL streptomycin (all from Gibco; Thermo Fisher Scientific, Inc., Waltham, MA, USA) within a humidified atmosphere containing 5% CO_2_ at 37 °C.

### Luciferase reporter assay

A luciferase test was done to see if MALAT1 and miR-561 had any direct interactions. The miR-561 binding sites in the section of MALAT1 were amplified by PCR and cloned into the Renilla luciferase reporter site of psiCHECK2 vectors. DNA sequencing was also used to ensure that the cloning process worked. For the luciferase experiment, MCF-7 and MDA-MB-231 cells were transfected with the psiCHECK-2 plasmid, which has positions 174–179 of MALAT1 with or without binding site changes, a negative control, and a miR-561 mimic.The Dual-Luciferase Reporter Assay System was used to conduct the luciferase assays 48 h following transfection in line with the manufacturer's instructions (Promega). A Synergy HTX microplate reader (BioTek, Winooski, VT, USA) was used to quantify the activity of Renilla luciferase, and the data were then normalized using the activity of Firefly luciferase.

### Targeting of MALAT1 by siRNA and cell transfection

A synthetic hsa-miR-561-3p mimic (Genolution, Seoul, Korea) was designed according to information registered in the miRBase database. A miRNA inhibitor targeting hsa-miR-561 (5′-UGG AAG ACU AGU GAU UUU GUU GUU-3′) and two siRNAs targeting MALAT1 (5′-CUA CAU UUG AGC AUA GUA U-3′) were synthesized by Bioneer Co. (Bioneer, Daejeon, Korea). Cell transfection was performed using Hiperfect Transfection Reagent (QIAGEN, Germany) according to the manufacturer’s instructions. The MALAT1 level was then confirmed 48 h later using RT-qPCR.

### RT-qPCR analysis of MALAT1, miRNA-561, and TOP2A

Total RNA was isolated from the prepared tissues and cells using the TRIzol reagent (Invitrogen). Complementary DNA was synthesized using the PrimeScript RT reagent kit (Takara, Dalian, China). qPCR was then performed using the fluorescent SYBR green master mix (Life Technology, 4309155). For miRNA, the primers and stem-loop were designed based on previously described methods, and U6 was used as the reference gene (Table 2). The data were calculated using the 2^−ΔΔCt^ method, and GAPDH was used as an endogenous control.

**Table 2:** Relative primers used for PCR, real-time PCR, stem-loop sequences.

Name	Sequences	
PCR analysis (3′UTR)		
Top2A	Sense Anti-sense	5′ CCGCTCGAGACCAAGCCCAAGACTGGTTT 3′ 5′ ATAAGAATGCGGCCGCGCACATAAGAGGCTGAGTGT 3′
MALAT1	Sense Anti-sense	5′ CCGCTCGAGTTCAGTTGTGTGTAAGCAAGT 3′ 5′ ATAAGAATGCGGCCGCAGATAATGTTCTCATCAGTAGTAAG 3′
qPCR primer		
Top2A	Sense Anti-sense	5′ CATTGAAGACGCTTCGTTATGG 3′ 5′ CAGAAGAGAGGGCCAGTTGTG 3′
MALAT1	Sense Anti-sense	5′ CTTCCCTAGGGGATTTCAGG 3′ 5′ GCCCACAGGAACAAGTCCTA 3′
miR-561	Sense Anti-sense	5′ CGCTCCAAAGTTTAAGATCCTTGAAG 3′ 5′ AGACTGCACCTGTCCGG 3′
GAPDH	Sense Anti-sense	5′ GAAAGCCTGCCGGTGACTAA 3′ 5′ GCGCCCAATACGACCAAATC 3′
U6	Sense Anti-sense	5′ CGCAAGGATGACACGCAAATTC 3′ 5′ AGACTGCACCTGTCCGG 3′
siRNA and mimic sequences		
siRAN1#MALAT1		5′-GGCUUAUACUCAUGAAUCUTT-3′
siRAN2#MALAT1		5′-GGGCUUCUCUUAACAUUUATT-3′
miR-561	Sense Anti-sense	5′ CAAAGUUUAAGAUCCUUGAAGU 3′ 5′ ACUUCAAGGAUCUUAAACUUUG 3′
Negative control		UUCUCCGAACGUGUCACGUTT
miRNA inhibitor negative control		CAGUACUUUUGUGUAGUACAA
miR-561 stem-loop		GTCGTATCCAGTGCAGGGTCCGAGGTATTCGCACTGGATACGACACTTCA

### Proliferation assessment by MTT assay

Using the MTT (3-(4,5-dimethylthiazol-2-yl)-2,5-diphenyltetrazolium bromide) test, the proliferation of BC cells was examined. BC cells were infused into 96-well plates at a density of 3 × 10^3^ cells per well 72 h after transfection. By pouring 20 L of a 5 mg/mL MTT solution (Sigma-Aldrich; Merck KGaA, Darmstadt, Germany) into each well, cell proliferation was assessed 72 h after seeding. For an additional 4 h, the plates were incubated at 37 °C in a humidified environment composed of 95% air and 5% CO_2_. Dimethyl sulfoxide (150 L) was used in place of the culture medium (Sigma-Aldrich; Merck KGaA, Darmstadt, Germany). Finally, an ELISA microplate reader was used to measure the absorbance at a wavelength of 490 nm (Bio-Rad Laboratories, Inc., Hercules, CA, USA).

### Cell cycle and apoptosis analysis

The cell cycle and apoptosis were determined as previously described^34^. After 48 h of siRNA transfection, cells were fixed with 70% ethanol, incubated with 1 mg/mL PI staining solution containing RNaseA (50 U/mL; Sigma) for 30 min, and DNA content was measured using a flow cytometer. For apoptosis analysis, cells were fixed and incubated with PI and Annexin-V-fluorescein isothiocyanate (FITC) in the dark. Stained cells were analyzed by flow cytometry. In each experiment, 20,000 cells were recorded every 200 s, and each sample was run in triplicate.

### Statistical analysis

GraphPad Prism 8.0 and SPSS version 18.0 were both utilized throughout every statistical analysis that was carried out. Each experiment is a representative sample of a minimum of three other separate studies. The information is provided as the mean together with the standard deviation (SD). A Student's t-test or a one-way analysis of variance was used to determine whether or not there were significant differences between the experimental groups. It was determined that a probability threshold of 0.05 was statistically significant, and the two-sided P values were calculated accordingly.

All methods were performed according to appropriate guidelines.

### Ethics approval and consent to participate

The present study was conducted under the instructions accepted by the Ethics Committee of Pasteur Institute of Iran (Ethical code: IR.PII.REC.1398.023), written informed consent to participate, and consent to publish forms was obtained from all participants involved in the present study.

### Informed consent

Written informed consent was obtained from all enrolled subjects.

## Supplementary Information

Supplementary Information.

## Data Availability

All data generated or analyzed during this study are included in this published article.
