# Supplementary material for: Targeting long non-coding RNA MALAT1 reverses cancerous phenotypes of breast cancer cells through microRNA-561-3p/TOP2A axis
Source: Sci Rep. 2023 May 27;13:8652. doi: 10.1038/s41598-023-35639-x (PMC10224942; doi:10.1038/s41598-023-35639-x)
Supplement: Supplementary file 1 — Supplementary Information. [file 41598_2023_35639_MOESM1_ESM.pdf]

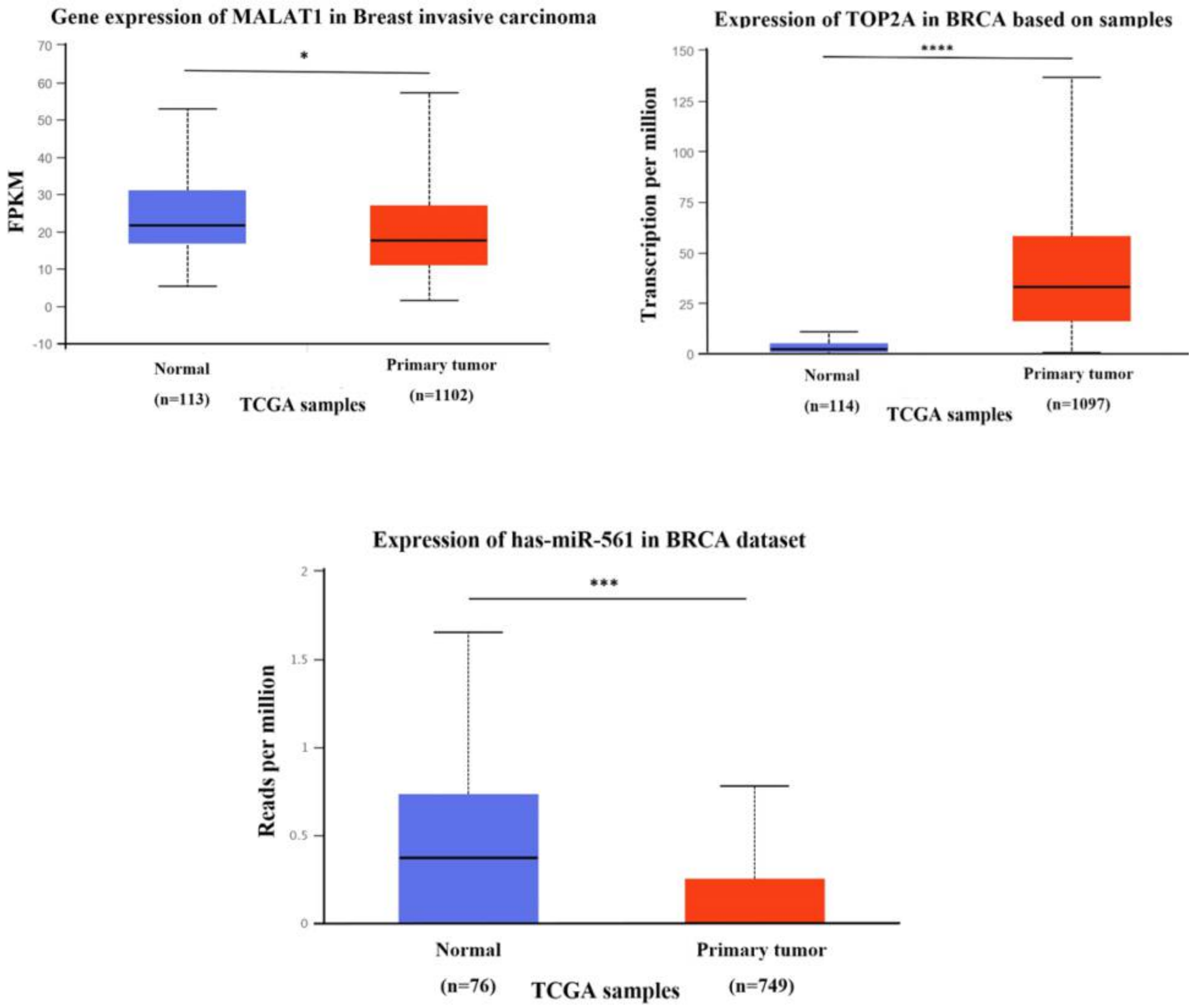
Gene expression of MALAT1 in Breast invasive carcinoma
FPKM
70
60
50
40
30
20
10
0
-10
*
Normal
(n=113)
TCGA samples
Primary tumor
(n=1102)
Expression of TOP2A in BRCA based on samples
Transcription per million
150
125
100
75
50
25
0
****
Normal
(n=114)
TCGA samples
Primary tumor
(n=1097)
Expression of has-miR-561 in BRCA dataset
Reads per million
2
1.5
1
0.5
0
***
Normal
(n=76)
TCGA samples
Primary tumor
(n=749)
